# Distribution of sequence types and antimicrobial resistance of clinical *Pseudomonas aeruginosa* isolates from dogs and cats visiting a veterinary teaching hospital in Thailand

**DOI:** 10.1186/s12917-024-04098-5

**Published:** 2024-05-31

**Authors:** Arunee Jangsangthong, Kittitat Lugsomya, Sukanya Apiratwarrasakul, Nathita Phumthanakorn

**Affiliations:** 1 Department of Pre-clinic and Applied animal science, Faculty of Veterinary Science, Mahidol University Salaya Campus, 999 Phutthamonthon Sai 4 Road Salaya, Phutthamonthon Nakhon Pathom, 73170 Thailand; 2 School of Veterinary Medicine, Murdoch University, Perth, WA Australia; 3 Veterinary Diagnostic Center of the Faculty of Veterinary Science, Mahidol University, 999 Phutthamonthon Sai 4 Road Salaya, Nakhon Pathom, Thailand

**Keywords:** Antimicrobial resistance, Cats, Sequence typing, Dogs, *Pseudomonas aeruginosa*

## Abstract

**Background:**

*Pseudomonas aeruginosa* is an important opportunistic pathogen in dogs and cats and is resistant to several antimicrobial drugs; however, data on the clonal distribution of *P. aeruginosa* in veterinary hospital are limited. This study aimed to investigate the clonal dissemination and antimicrobial resistance of clinical *P. aeruginosa* in a veterinary teaching hospital in Thailand within a 1-year period. Minimum inhibitory concentration determination and whole genome sequencing were used for antimicrobial susceptibility analysis and genetic determination, respectively.

**Results:**

Forty-nine *P. aeruginosa* were isolated mostly from the skin, urinary tract, and ear canal of 39 dogs and 10 cats. These isolates belonged to 39 sequence types (STs) that included 9 strains of high-risk clones of ST235 (*n* = 2), ST244 (*n* = 2), ST274 (*n* = 2), ST277 (*n* = 1), ST308 (*n* = 1), and ST357 (*n* = 1). Overall antimicrobial resistance rate was low (< 25%), and no colistin-resistant strains were found. Two carbapenem-resistant strains belonging to ST235 and ST3405 were identified.

**Conclusions:**

Clinical *P. aeruginosa* in dogs and cats represent STs diversity. High-risk clones and carbapenem-resistant strains are a public health concern. Nevertheless, this study was limited by a small number of isolates. Continuous monitoring is needed, particularly in large-scale settings with high numbers of *P. aeruginosa*, to restrict bacterial transfer from companion animal to humans in a veterinary hospital.

**Supplementary Information:**

The online version contains supplementary material available at 10.1186/s12917-024-04098-5.

## Background

*Pseudomonas aeruginosa* is a ubiquitous bacterium that can survive in various environment conditions [1]. It is an important opportunistic and nosocomial pathogen in humans and is associated with cystic fibrosis, burn wound infection, ulcerative keratitis, and other infections [1, 2]. In companion animals, *P. aeruginosa* is commonly isolated from the skin, ears, eyes, and infected urinary tract; thus, affecting animal health and welfare [3–5]. Although *P. aeruginosa* does not often cause a life-threatening disease in companion animals, treating this pathogen is difficult when it develops the resistance to several antimicrobial agents due to various intrinsic and acquired resistance mechanisms [5, 6]. The occurrence of multidrug-resistant (MDR) and carbapenem-resistant *P. aeruginosa* and the co-occurrence of antimicrobial resistance genes in isolates from companion animals have been reported [4, 7, 8]. Companion animals, especially dogs and cats, could be a bacterial reservoir, and transfer *P. aeruginosa* or its resistance gene to humans who are in close contact and vice versa [8–11].

The clonal distribution of *P. aeruginosa* isolates from humans, animals, and environment is nonspecific and diverse [12]. The most important clones are those of high-risk determined through multilocus sequence typing (MLST) e.g., ST235, ST111, ST244, ST357, ST308, ST175, ST277, and ST654 [13]. These high-risk clones are distributed worldwide and can be MDR and extensively drug-resistant, which is associated with treatment failure [14]. In companion animals, a few molecular epidemiology studies of clinical *P. aeruginosa* were conducted [3, 4, 7, 8, 10, 15]. High-risk clones were observed in carbapenem-resistant *P. aeruginosa* isolates from dogs in Japan [8] and France [7]. Furthermore, our previous study identified the presence of MDR and carbapenem-resistant *P. aeruginosa* strains belonging to ST235, ST244, and ST606 in the environment of a veterinary teaching hospital in Thailand [16]. However, data are lacking on the sequence types and the prevalence of high-risk clones of *P. aeruginosa* that cause infection in dogs and cats in the veterinary hospital in Thailand.

This study aimed to investigate the sequence typing and clones, and antimicrobial resistance of clinical *P. aeruginosa* isolates from the dogs and cats visiting a veterinary teaching hospital in Thailand within a 1-year period. Whole genome sequencing and analysis were performed to gain a deep perspective of intrahospital clonal dissemination.

## Results

### Clinical characteristic of *P. aeruginosa*

In 2022, 1,627 samples were collected from sick dogs and cats visiting Prasu-Arthorn Veterinary Teaching Hospital, Mahidol University, Thailand. Positive bacterial cultures were prepared from 821 samples that included nonduplicate 49 *P. aeruginosa* (6%, 49/821). These samples were obtained from 39 dogs (79.6%, 39/49) and 10 cats (20.4%, 10/49). The most frequent isolation site was skin wound (32.7%), followed by urinary tract (28.6%) and ear canal (24.5%) (Table 1 and Additional file 1). The other sites were nasal cavity and abdominal cavity (4.1% each), as well as the oral cavity, reproductive tract, and pleural cavity (2.1% each). In dogs (*n* = 39), skin wound and pus on skin (35.9%) were the most frequent isolation sites. In cats, *P. aeruginosa* was equally isolated from the skin, ear canal, urinary tract, abdominal cavity, and nasal cavity (20%, *n* = 2 each) (Table 1).

**Table 1:** Number and percentage of *Pseudomonas aeruginosa* isolates from different sampling sites

Sampling sites	Dogs (*n* = 39)		Cats (*n* = 10)		Total (*n* = 49)			
	*n*	%	*n*	%		*n*	%	
Skin (wound, pus)	14	35.9	2	20.0		16	32.7	
Urinary tract (urine)	12	30.8	2	20.0		14	28.6	
Ear canal	10	25.6	2	20.0		12	24.5	
Abdominal cavity	1	2.6	1	10.0		2	4.1	
Nasal swab	0	0	2	20.0		2	4.1	
Oral cavity	1	2.6	0	0		1	2.1	
Reproductive tract	1	2.6	0	0		1	2.1	
Pleural cavity	0	0	1	10.0		1	2.1	

### Distribution of STs of clinical ***P. aeruginosa***

**Fig. 1:**
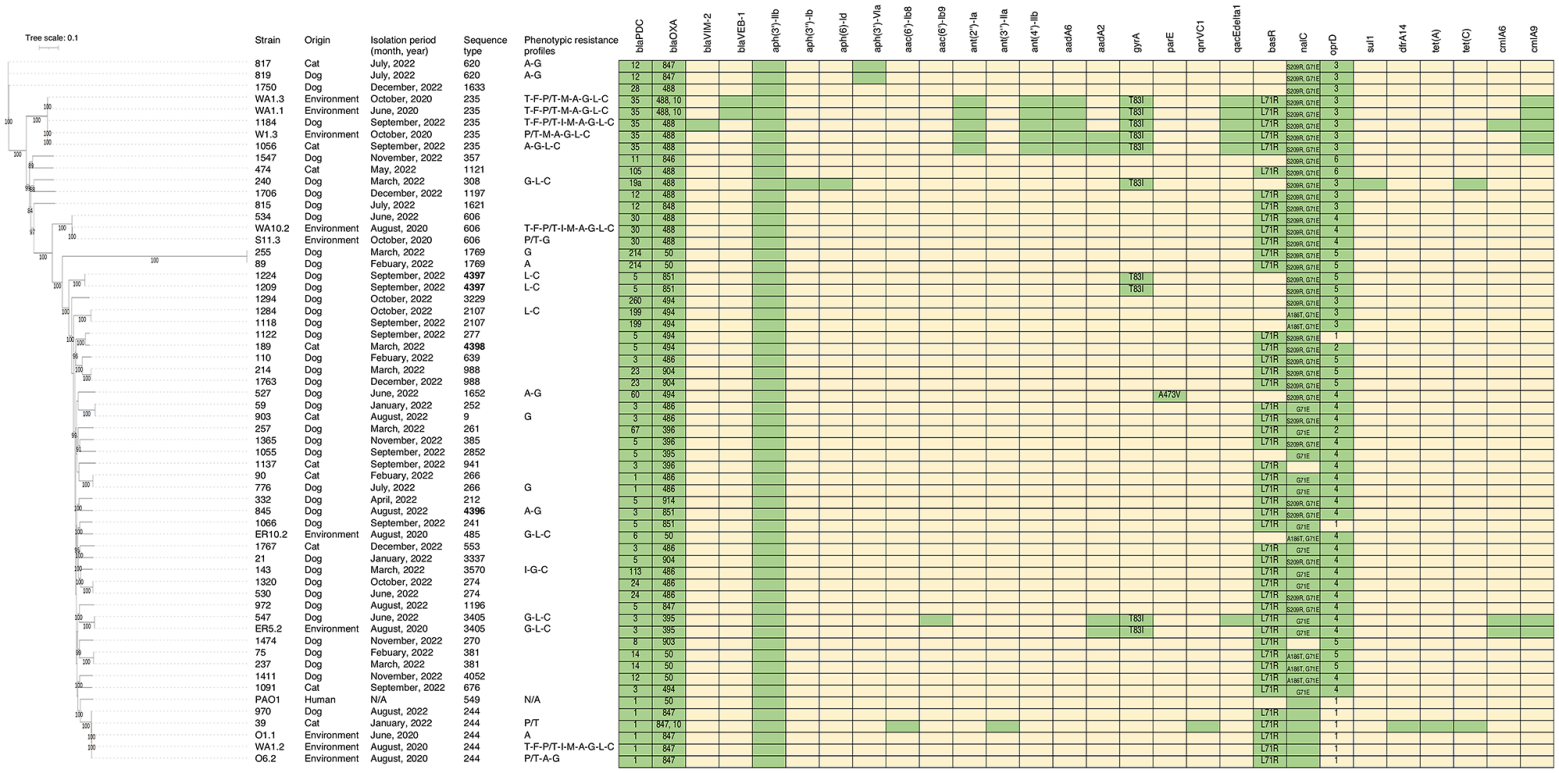
Phylogenetic single nucleotide polymorphism (SNP) tree, strain, sequence types (STs), origin, isolation period, and antimicrobial resistance of *Pseudomonas aeruginosa*. Ten environmental isolates from a previous study [16] and the reference strain *P. aeruginosa* PAO1 (Accession no. AE004091.2) were included. New ST in bold. Presence in green and absence in yellow. N/A, not determined. T, cefazidime, F, cefepime, P/T, piperacillin/ tazobactam, I, imipenem, M, meropenem, A, amikacin, G, gentamicin, L, Levofloxacin, C, ciprofloxacin, and in blank, susceptible. Types of *bla*_PDC_ and *bla*_OXA_; and amino acid mutation of *gyrA*, *parE*, *basR*, *nalC* in green box. *oprD* amino acid alterations: 1, wildtype; 2, T103S, K115T, F170L; 3, T103S, K115T, F170L, E185Q, P186G, V189T, R310E, A315G, G425A; 4, D43N, S57E, S59R, E202Q, I210A, E230K, S240T, N262T, A267S, A281G, K296Q, Q301E, R310G, V359L, 372VDSSSS–YAGL383; 5, V127L, E185Q, P186G, V189T, E202Q, I210A, E230K, S240T, N262T, T276A, A281G, K296Q, Q301E, R310E, R310E, G312R, A315G, L356M, L348M, 372VDSSSS–YAGL383, S403A, Q424E; 6, S57E, S59R, V127L, E185Q, P186G, V189T, E202Q, I210A, E230K, S240T, N262T, T276A, A281G, K296Q, Q301E, R310E, A315G, L347M, 372VDSSSS–YAGL383, S413A, Q424E

*P. aeruginosa* belonged to 39 STs, which included 3 new STs: ST4396 (*n* = 1), ST4397 (*n* = 2), and ST4398 (*n* = 1) distributed in this 1-year study (Fig. 1). No predominant clones were determined from the STs; only one or two *P. aeruginosa* strains were included in the clones. Total of 9 strains (18.37%) belonged to the 6 high-risk clones of ST235 (*n* = 2), ST244 (*n* = 2), ST274 (*n* = 2), ST277 (*n* = 1), ST308 (*n* = 1), and ST357 (*n* = 1) (Fig. 1). These strains were isolated from various sampling sites including skin (ST235 and ST308), ear canal (ST235, ST244, and ST357), urinary tract (ST244, ST274, and ST277), and abdominal cavity (ST244) (Additional file 1). Additionally, the 6 high-risk clones were isolated during various isolation periods (Fig. 1). The isolates from the same STs were closely clustered together, according to phylogenetic SNP tree and SNP number analyses of clinical isolates from this study and 10 environmental isolates from a prior study conducted at the same veterinary hospital (Fig. 1 and Additional file 2).

### Antimicrobial resistance of ***P. aeruginosa***

Among the *P. aeruginosa* isolates, 16 were resistant to at least one of the drugs tested and 33 (67.4%) were susceptible strains. For the 16 strains, the highest phenotypic resistant rate was observed for gentamicin (24.5%), followed by amikacin (14.3%), ciprofloxacin (14.3%), and levofloxacin (12.2%). Resistance to piperacillin/ tazobactam and imipenem accounted for 4.1% each, and resistance to ceftazidime, cefepime, and meropenem was at 2%. No colistin resistance was observed. Two MDR strains (1184 and 143) were identified (2%, 2/49) and found to be carbapenem-resistant strains. Strain 143 was resistant to imipenem, gentamicin, and ciprofloxacin; and strain 1184 was resistant to ceftazidime, cefepime, piperacillin/tazobactam, imipenem, meropenem, gentamicin, amikacin, levofloxacin, and ciprofloxacin (Fig. 1).

Genes responsible for acquired and phenotypic antimicrobial resistance and those with amino acid alterations were selected from the Comprehensive Antibiotic Resistance Database (CARD) database for analysis in Fig. 1. In general, the strains belonging to the same STs exhibited a similar antimicrobial resistance gene profile, except for ST235 and ST244. All of them harbored different types of β-lactamases, *bla*_PDC_ and *bla*_OXA_ (Fig. 1). The combination of *bla*_VIM−2_, *bla*_PDC−35_ and *bla*_OXA−488_ was detected in a single strain 1184-ST235 that was resistant to imipenem and meropenem. While two carbapenem-resistant ST235 isolates from the environment (strains WA1.3 and WA1.1) contained *bla*_VEB−1_, *bla*_PDC−35_, *bla*_OXA−488_, and *bla*_OXA−10_ (Fig. 1). Aminoglycoside resistance gene *aph(3’)-Ilb* was detected in all the isolates alone or in combination with the others. The gentamicin and/or amikacin resistance genes were *aph(3’’)-Ib*, *aph(6)-Id* in ST308, *ant(2’’)-Ia*, *ant(3‘’)-Ila*, *ant(4’)-Ilb*, *aadA6*, and *aadA2* in ST235; *aph(3’)-Vla* in ST620; and *aac(6’)-Ib9* and *aadA2* in ST3405. A single strain ST244 harboring *aac(6’)-Ib8* and *ant(3‘’)-Ila* was susceptible to gentamicin and amikacin. Except for *aph(3’)-Ilb*, none of the aminoglycoside resistance genes were found in the seven gentamicin- and/or amikacin-resistant strains. The mutation in *gyrA* (T83I) but not in *parE* (A473V) or *qnrVC1* was associated with resistance to ciprofloxacin and levofloxacin in clinical isolates. The disinfectant resistance gene, *qacEdelta1*, was identified in two strains of ST235 and a single strain 547-ST3405. A sulfonamide resistance gene (*sul1*) and a tetracycline resistance gene [*tet*(C)] were found in strain 240-ST308, and trimethoprims (*dfrA14*) and *tet*(A) were found in strain 39-ST244. Similar to strain 1184-ST235, strain 39-ST244 also contained the chloramphenicol resistance genes, *cmlA6* and *cmlA9*. A point mutation of *basR* (L71R) gene associated with polymyxcin B resistance was observed at 75.5% (*n* = 37). None of *nalC* hit search (*n* = 2), wild type (*n* = 2), and mutation in the *nalC* gene of efflux pump regulatory genes at G71E (*n* = 11), A186T/G71E (*n* = 5), and S209R and G71E (*n* = 29) were found. Among the 49 strains, 45 had several polymorphisms of the amino acid alterations of *oprD* (Fig. 1).

## Discussion

The several STs with genetic diversity of clinical *P. aeruginosa* disseminated in a veterinary teaching hospital were discovered by MLST and whole genome analysis. Clinical *P. aeruginosa* was mainly isolated from the skin, urinary tract, and ear canal, which in consistent with a previous study but shows a different prevalence [17, 18]. The genomic diversity of *P. aeruginosa* was observed from different infection types (keratitis and cystic fibrosis) in humans [19]. However, no specific clone was found on each sampling or infection sites during MLST and phylogenetic SNP tree analysis in the present work.

Six high-risk clones of ST235, ST244, ST274, ST277, ST308, and ST357 circulated in the dogs and cats in this study. The dissemination of ST235 and ST244 was previously reported in the environment of the same veterinary hospital in 2020 [16]. ST244 is a frequently found clone but is not necessarily associate with antimicrobial resistance [13], which supports the findings in this recent study. Among them, ST235 is the most relevant and widespread high-risk clone worldwide; it produces different carbapenemases in association with horizontally acquired resistance and harbors high virulence due to the production of the potent type III secretion system exotoxin encoded by *exoU* gene [13]. In addition, carbapenem-resistant *P. aeruginosa* ST235 was the predominant clone in the clinical isolates of dogs and cats in Japan [8]. This recent study found that strain 1184-ST235 was resistant to β-lactams, carbapenem, aminoglycosides, and fluoroquinolone. Resistance to imipenem and meropenem in strain 1184-ST235 mediated by the VIM-2 metallo-β-lactamase (*bla*_VIM−2_) of *P. aeruginosa* is the most common metallo-β-lactamase gene in many regions of Thailand and Asia [4, 20, 21]. Meanwhile, ST235 isolates from the environment of the same veterinary hospital contained *bla*_VEB−1_ [16]. Therefore, ST235 has high potential to be distributed in the dogs and cats and environment of the veterinary hospital with different antimicrobial resistance levels.

Apart from the *bla*_VIM−2_ gene, several types of β-lactamase genes *bla*_PDC_ and *bla*_OXA_ were identified in all the isolates. PDC and OXA types are the predominant chromosomal β-lactamase in *P. aeruginosa* [22]. The different combinations of *aph(3’’)-Ib*, *aph(6)-Id*, *aadA2*, *aadA6*, *qacEdelta1*, *dfrA14*, *sul1*, *cmlA6*, and *cmlA9* could represent the integron class I [14, 19] in strains 240-ST308, 1056-ST235, 1184-ST235, and 547-ST3405. Integron class I with diverse gene cassette array is common in *P. aeruginosa*, especially in the high-risk clones, and is associated with horizontally acquired antimicrobial resistance gene and MDR [14, 20].

The overall resistance rate in this study was low (< 25%). The resistance rate of gentamicin (24.5%) was the highest rate, which was also discovered in a previous investigation of *P. aeruginosa* isolate from dogs in Korea (26.3%) [4] and the isolates from the environment in the same veterinary hospital (47.4%). Gentamicin is frequently used as a topical drug to treat otitis externa and skin infection in this veterinary hospital, and this practice could influence its resistance. The resistance rate to carbapenem (4.1%), including imipenem (4.1%) and meropenem (2%), was lower in the current work than that in dogs in Korea (imipenem 12.5% and meropenem 16.3%) [4], animals in France (5.7%) [7], dogs and cats across Japan (imipenem 6.7% and meropenem 2.1%) [8]. Carbapenem is preserved and restrict to treat severe infection in the veterinary teaching hospital in the present study. Although the carbapenem resistance rate was low, the increasing prevalence of carbapenem-resistant *P. aeruginosa* has been reported in other monitoring studies [7, 8]. Therefore, the continuous monitoring of MDR and carbapenem-resistant is warranted, especially in the veterinary hospital in many regions of Thailand. With regard to the absence of genes for the phenotypic resistance of a single imipenem-resistant strain and seven amikacin- and/ or gentamicin-resistant strains, other mechanisms, including the overexpression of efflux pumps e.g., MexXY and MexEF-OprN, and the accumulation of multiple mutations, may be responsible for this antimicrobial resistance [6, 14].

## Conclusion

Diverse STs of clinical *P. aeruginosa* were disseminated in a veterinary teaching hospital in Thailan during a 1-year period. Six high-risk clones of ST235, ST244, ST274, ST277, ST308, and ST357 were observed without predominance and specificity to the sampling sites. This study revealed the low antimicrobial resistance rate, including carbapenem resistance and MDR. The limitation of this study included the small number of isolates from a single veterinary hospital. The expanded scale of monitoring and surveillance in more than one veterinary hospital is necessary to assess the situation and design the prevention strategy for veterinary medicine.

## Methods

### Bacterial isolates

*P. aeruginosa* was isolated from various infection sites of sick dogs and cats visiting Prasu-Arthorn Veterinary Teaching Hospital, Mahidol University, Thailand during January–December 2022. The samples were collected by the veterinarians and were sent for routine species identification at the Veterinary Diagnostic Center of the Faculty of Veterinary Science, Mahidol University, Thailand. The samples were cultured on tryptic soy agar (TSA) with 5% sheep blood (Biomedia, Thailand) and MacConkey agar (Biomedia, Thailand), and were incubated at 35 ± 2 °C for 24 h. Single colony was subcultured on TSA with 5% sheep blood agar and incubated at 35 ± 2 °C for 24 h in order to obtain homogeneous colonies. The species identification was performed using biochemical tests, including oxidase test, citrate test, motility tests, triple sugar iron reaction, and production of yellow-green or blue-green diffusible pigment on King A and King B media. The isolates identified as *P. aeruginosa* were included in this study. Their species were confirmed by MALDI-TOF MS (Bruker Daltonics, Germany).

### Antimicrobial susceptibility testing

The minimum inhibitory concentration (MIC) for 10 antipseudomonal drugs was determined using THN1F Sensititre broth microdilution following the manufacturer’s recommendations (Sensititre, Thermo Scientific, Waltham, MA, USA). The breakpoints for ceftazidime, piperacillin/tazobactam, imipenem, levofloxacin, amikacin, and gentamicin were interpreted following the guidelines available for animals [23], and the breakpoints used for cefepime, tetracycline, meropenem, ciprofloxacin, and colistin were interpreted following the breakpoints for humans [24]. The *P. aeruginosa* isolates were grown on 5% sheep blood agar and incubated at 37 °C for 24 h before use. *Escherichia coli* ATCC 25922 was used as positive control for MIC determination. MDR was defined as the isolate being resistant to at least one agent in ≥ 3 classes of the antimicrobial drugs tested [25]. The intermediate interpretation was considered as susceptible following the definition of susceptibility testing categories by EUCAST (www.eucast.org).

### Whole genome sequencing and analysis

Genomic DNA was extracted using commercial kits (ZymoBiomics DNA miniprep kit, ZymoResearch, USA) following the manufacturer’s instructions. The genomes were sequenced using Illumina NovaSeq 6000 (150 bp paired end, Illumina, San Diego, USA), and the raw reads were processed using fastp v.0.23.3 [26]. *De novo* assembly was performed using SPAdes in Shovill v.1.1.0 [27, 28]. The contigs size of ≥ 200 bp were used for gene annotation in Prokka v.1.14.6 [29]. The antimicrobial resistant genes were identified using the CARD [30]. The sequence of the *oprD* gene was compared with the reference strain *P. aeruginosa* PAO1 (Accession no. AE004091.2) using Clustal Omega of EMBL-EBI website (https://www.ebi.ac.uk/Tools/msa/clustalo/). MLST was analyzed for sequence types (STs) in pubMLST website (https://pubmlst.org) [31]. The phylogenetic tree of *P. aeruginosa* was constructed from the concatenated sequence of core-single nucleotide polymorphisms (core-SNPs) located in the region of core-genome of 49 *P. aeruginosa* from this study, with *P. aeruginosa* strain PAO1 as the reference strain (accession no. AE004091), and 10 environmental isolates from the previous study [16]. The core sequences were aligned by Parsnp program with the default parameter settings [32], and the recombination regions were filtered off using Gubbins [33]. The SNP sites were then called and concatenated by snippy (https://bactopia.github.io/bactopia-tools/snippy/). The phylogenetic tree was constructed using the maximum likelihood method with the GTR + Γ substitution model and 1,000 bootstrap replicates in the IQTREE 2 program [34]. The tree was visualized with Itol v6 online version (https://itol.embl.de/) [35].

### Electronic supplementary material

Below is the link to the electronic supplementary material.

Supplementary Material 1

Supplementary Material 2

## Data Availability

This whole genome project has been deposited at GenBank of NCBI under bioproject PRJNA1001300. The accession are JAUZAE000000000- JAUZAZ000000000, JAUZBA000000000-JAUZBZ000000000, and JAVCNP000000000. The genomic data of this article is available in GenBank of NCBI repository, https://www.ncbi.nlm.nih.gov/bioproject/?term=PRJNA1001300. The datasets supporting the conclusions of this article are included within the article and its additional file 1 and additional file 2.

## Abbreviations

P. aeruginosa: Pseudomonas aeruginosa
MLST: Multilocus sequence typing
ST: Sequence typing
VIM-2: Verona imipenemase-2
MALDI-TOF: MS Matrix-Assisted Laser Desorption/Ionization Time-of-Flight Mass Spectrometer
CLSI: Clinical & Laboratory Standards Institute
EUCAST: European Committee on Antimicrobial Susceptibility Testing
ATCC: American Type Culture Collection
SNP: Single nucleotide polymorphism
CARD: Comprehensive Antibiotic Resistance Database
