# Supplementary material for: Distribution of sequence types and antimicrobial resistance of clinical Pseudomonas aeruginosa isolates from dogs and cats visiting a veterinary teaching hospital in Thailand
Source: BMC Vet Res. 2024 May 31;20:234. doi: 10.1186/s12917-024-04098-5 (PMC11140974; doi:10.1186/s12917-024-04098-5)

Additional file 2: Phylogenetic single nucleotide polymorphism (SNP) and SNP number of 49 *Pseudomonas aeruginosa.* Ten environmental isolates from a previous study [16] and the reference strain *P. aeruginosa* PAO1 (Accession no. AE004091.2) were included. Additional file 2.tiff


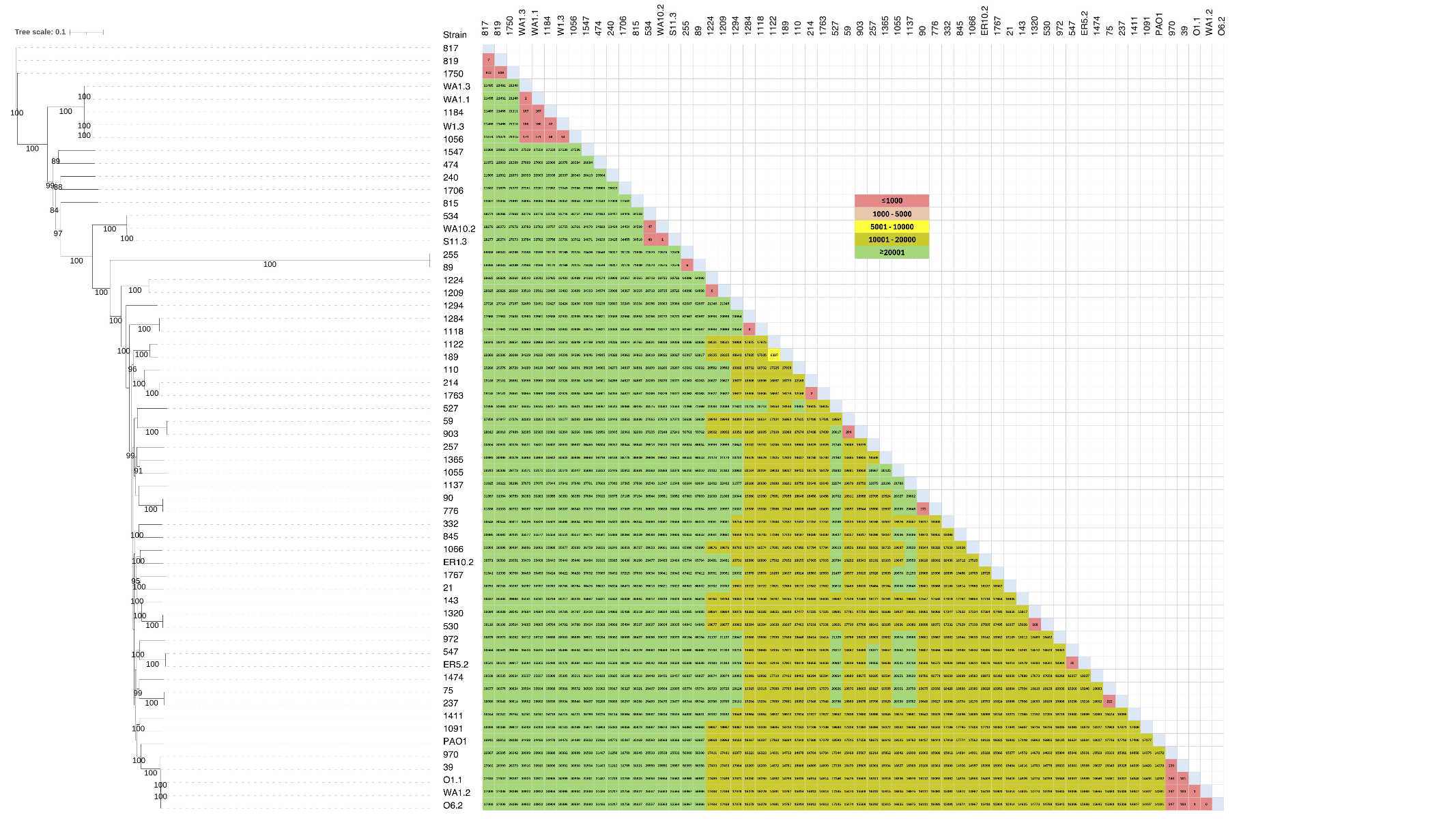

Supplement: Supplementary file 1 — Supplementary Material 1 [file 12917_2024_4098_MOESM1_ESM.docx]
